# “Am I a candidate for magic mushrooms?” A mystery shopper study of Oregon psilocybin service centers

**DOI:** 10.1093/haschl/qxag231

**Published:** 2026-08-27

**Authors:** Danielle R Adams, Morgan Hartman, Lisa Gong, Suraj Shankar, Ruth Ling, Jacob T Steinle, Joshua S Siegel, Ginger E Nicol, Richard A Grucza, Jacob T Borodovsky, Leopoldo J Cabassa, Kevin Y Xu

**Affiliations:** School of Social Work, College of Health Sciences, University of Missouri, Columbia, MO 65201, United States; Bursky School of Public Health, Washington University, St Louis, MO 63110, United States; George Warren Brown School of Social Work, Washington University, St Louis, MO 63130, United States; Center for Advancing Health Services, Policy and Economics Research, Washington University, St Louis, MO 63110, United States; Center for Advancing Health Services, Policy and Economics Research, Washington University, St Louis, MO 63110, United States; Department of Psychiatry, Washington University School of Medicine, St Louis, MO 63110, United States; Center for Advancing Health Services, Policy and Economics Research, Washington University, St Louis, MO 63110, United States; Department of Psychiatry, Washington University School of Medicine, St Louis, MO 63110, United States; Department of Psychiatry, Icahn School of Medicine at Mount Sinai, New York, NY 10029, United States; Division of Child and Adolescent Psychiatry, Massachusetts General Hospital, Harvard Medical School, Boston, MA 02114, United States; Department of Psychiatry, NYU Grossman School of Medicine, New York, NY 10016, United States; Nathan S. Kline Institute for Psychiatric Research, Orangeburg, NY 10962, United States; Department of Psychiatry, Washington University School of Medicine, St Louis, MO 63110, United States; Center for Holistic Interdisciplinary Research in Psychedelics, Washington University, St Louis, MO 63110, United States; Advanced Health Data Institute, Department of Health and Clinical Outcomes Research and Department of Family and Community Medicine, Saint Louis University, St Louis, MO 63104, United States; The Dartmouth Institute for Health Policy and Clinical Practice, Dartmouth Geisel School of Medicine, Lebanon, NH 03756, United States; George Warren Brown School of Social Work, Washington University, St Louis, MO 63130, United States; Center for Holistic Interdisciplinary Research in Psychedelics, Washington University, St Louis, MO 63110, United States; Bursky School of Public Health, Washington University, St Louis, MO 63110, United States; Center for Advancing Health Services, Policy and Economics Research, Washington University, St Louis, MO 63110, United States; Department of Psychiatry, Washington University School of Medicine, St Louis, MO 63110, United States

**Keywords:** psilocybin, psychedelics, psychedelic assisted therapy, health services accessibility, health policy, Oregon, mental health services, patient safety, health communication, health care costs, secret shopper, magic mushrooms, drug policy, mystery shopper

## Abstract

**Introduction:**

Oregon is the first US state to permit supervised psilocybin services through a nonmedical, wellness-based model. We examined what prospective clients with psychiatric histories encounter when contacting service centers, including how staff communicate about safety, eligibility, and cost.

**Methods:**

We conducted a mystery shopper study of 26 licensed service centers in Oregon (7/2025-9/2025), 4 of which closed before calls began, leaving 22 in the sample. We reviewed websites and conducted 2 waves of scripted phone calls portraying adults with depression. Website and call data were combined at the center level, counting an item as disclosed if it appeared online or during a call. Themes from call field notes were contextualized through structured debriefing interviews with service center staff.

**Results:**

Callers reached 15/22 centers, none of which permitted unsupervised consumption or immediate dosing without psychiatric assessment. Staff asked detailed questions about psychiatric comorbidities, addressing information gaps from websites, and none recommended discontinuing antidepressants. The median cost was $1900. Debriefing interviews described financial strain for service centers, with viability constrained by licensing fees and potential misalignment with cannabis-derived business models.

**Conclusion:**

Oregon's regulated model appears clinically cautious, while facing high costs for clients, facilitators, and service centers that limit scalability.

## Introduction

Psychedelic policy is evolving rapidly in the United States,^1,2^ evidenced by an April 2026 executive order directing the FDA to prioritize review of psychedelic compounds with Breakthrough Therapy designation for serious mental illness.^3^ This momentum comes as psilocybin (magic mushrooms) is already accessible throughout the United States via a patchwork of models that differ in how much supervision they require.^1,2^ Oregon was the first state to authorize supervised psilocybin use through state-licensed service centers in 2020, and Colorado followed shortly in 2022, developing a supervised psilocybin program in addition to permitting adults to possess, cultivate, and share psilocybin without payment under state law. Several municipalities (eg, Oakland, Seattle) have deprioritized enforcement for personal possession. Outside such pathways (along with clinical research, limited investigational access, and case-specific religious exemptions), psilocybin remains federally prohibited. How these models differ in safety, eligibility, and cost is poorly understood, particularly for clients with mental health conditions drawn to psilocybin for its therapeutic promise.^4-6^

Under the Oregon Health Authority (OHA) framework for supervised psilocybin services, the first of its kind in the United States,^7,8^ services are delivered as a wellness-based offering rather than medical treatment. Clients need no diagnosis, clinician referral, or medical clearance; facilitators screen at a preparation session for 3 mandatory exclusions (lithium use within the previous 30 days; current thoughts of harming oneself or others; diagnosis of or treatment for active psychosis; OAR 333-333-5050). Of the 3 service components (preparation, administration, and integration), only the first 2 are required; integration must be offered but may be declined (OAR 333-333-5130). Further, facilitators need no clinical degree or professional license, only that they be at least 21 with a high school diploma, complete OHA-approved training and a practicum, pass examinations, and clear a background check, though 57.3% of Oregon's first licensed cohort held a prior healthcare or mental health license.^9^ Service centers hold a separate license, with its own premises, security, and recordkeeping requirements.

While Oregon's service centers are prohibited from making deceptive claims or unsupported therapeutic representations,^10,11^ concerns have been raised that wellness-based psychedelic services may expose consumers to inconsistent advertising, unclear risk communication, or inadequate informed consent.^12^ Available evidence is limited but suggests variation in how psychedelic services screen clients and communicate risk. Stakeholders have described variation in screening practices and differing approaches to accountability across service settings,^13^ while an audit of Canadian psilocybin dispensary websites found frequent unsupported health claims and uneven disclosure of risks.^14^ Licensed centers also operate alongside an informal (“underground”) market outside the regulatory framework. No published evidence describes what prospective clients with psychiatric comorbidities actually encounter when they contact licensed centers.^5,6,13,15-19^ To address this gap, we conducted a mystery shopper study of Oregon's service centers, focusing on 3 policy-relevant questions for clients with psychiatric comorbidities: (1) how centers communicate about safety, including contraindications, medication guidance, and requests for unsupervised use; (2) how they make eligibility decisions when clients disclose risk-relevant histories; and (3) what costs these clients encounter.

## Methods

We conducted a cross-sectional convergent mixed-methods mystery shopper study^20^ of all licensed Oregon psilocybin service centers, triangulating data from website review, telephone calls, and debriefing interviews with service-center staff. Mystery shopper methods simulate real-world interactions and help overcome social desirability bias and idealized self-reports that limit announced or survey-based inquiries,^21^ making them well-suited to capture what prospective clients actually encounter. Our design thus mirrored the pathway a prospective client might take, beginning with website review and proceeding to telephone contact, after which service-center staff were debriefed by our study team on emerging themes from our data.^22^ The study was IRB approved via a waiver of prospective informed consent for the mystery shopper phase under minimal-risk criteria; verbal consent was obtained for the debriefing interviews.

### Service center sample identification

Centers were identified through the OHA public licensee directory,^23^ a principal channel through which centers are licensed and advertise to prospective clients, and thus a reasonable frame for the centers a client would encounter. To confirm the directory reflected the active service landscape,^14^ we triangulated it against additional sources: structured online searches (Google and Google Maps, using terms such as “psilocybin,” “psychedelic,” “magic mushroom,” and “shroom” combined with Oregon locations), psychedelic-specific directories (eg, psychedelic.support), and regional tourism websites (eg, visitbend.com), supplemented by snowball searches from these results and cross-checks with stakeholders familiar with Oregon's psilocybin service landscape. These searches identified no active centers beyond those listed in the directory.^24^

### Codebook

Before data collection, we developed a codebook (eMethods 2) informed by a published evidence review and client-information requirements (OAR 333-333-5050) from the OHA Psilocybin Advisory Board,^18^ a state-appointed body established under ORS 475A.225 whose membership includes state public health officials and representatives from medicine, public health, research, mycology, harm reduction, and indigenous communities. We did not rely on unpublished Board deliberations. The codebook operationalized the 3 audit domains (safety, eligibility, cost) across both website and call data, with explicit criteria for each variable refined iteratively as new response patterns emerged. The left side of Table 1 lists the criteria within each domain (eg, explicit mention of lithium under safety). Website items were coded as present or absent. Call responses involving screening or eligibility were coded as affirmative, equivocal, or negative. Cost and timing measures were recorded as continuous variables.

**Table 1.** Communication, eligibility, and access findings from website review and mystery shopper calls at Oregon psilocybin service centers.

Domain/Measure	Centers, n (%)
**Safety and Risk Assessment**	
**Website information vs clarification during calls**	
Psychiatric history required at intake	Website only: 15/15 (100%) During calls: 15/15 (100%) Combined: 15/15 (100%)
Age requirement/verification (21+)	Website only: 12/15 (80.0%) During calls: 7/15 (46.7%) Combined: 14/15 (93.3%)
Lithium articulated as contraindication	Website only: 7/15 (46.7%) During calls: 11/15 (73.3%) Combined: 14/15 (93.3%)
Active suicidal ideation articulated as contraindication	Website only: 9/15 (60.0%) During calls: 13/15 (86.7%) Combined: 15/15 (100%)
**Phone call: SSRI management**	
Continue SSRI	8/15 (53.3%)
Equivocal/Uncertain	7/15 (46.7%)
Taper SSRI	0/15 (0%)
**Phone call: “Will psilocybin help?” for depression** *n* = *15 centers*	
Yes	12/15 (80.0%)
Equivocal/Uncertain	3/15 (20.0%)
No	0/15 (0%)
**Phone call: “Is psilocybin safe for me?” (alcohol use disorder)** *Script 1, n* = *15 centers*	
Can be safe	12/15 (80.0%)
Equivocal/Uncertain	3/15 (20.0%)
Not safe	0/15 (0%)
**Phone call: “Is psilocybin safe for me?” (family history of psychosis)** *Script 2, n* = *12 centers*	
Can be safe	7/12 (58.3%)
Equivocal/Uncertain	5/12 (41.7%)
Not safe	0/12 (0%)
**Eligibility**	
**Phone call: “Am I a candidate?” (alcohol use disorder)** *Script 1, n* = *15 centers*	
Yes	9/15 (60.0%)
Equivocal/Uncertain	6/15 (40.0%)
No	0/15 (0%)
**Phone call: “Am I a candidate?” (family history of psychosis)** *Script 2, n* = *12 centers*	
Yes	8/12 (66.7%)
Equivocal/Uncertain	4/12 (33.3%)
No	0/12 (0%)
**Cost and access**	
**Phone call: Cost and access timing (*n*** **=** **15 centers)**	
Estimated cost, USD—median (range)	$1900 ($850–$3000)
Time to initial successful contact with service center, days—median (range)	2.0 (1-8.5)
Time to intake appointment, days—median (range)	9.25 (5-30)
**Phone call: Access characteristics (*n*** **=** **15 centers)**	
Health Savings Account accepted	4/15 (26.7%)
Insurance accepted	0/15 (0%)
Sliding scale offered	15/15 (100%)
Out-of-state clients accepted	15/15 (100%)

Website vs call values reflect information available to a prospective client across a typical access pathway. “On websites” indicates information publicly available online among reached centers (*n* = 15). “Website followed by phone call” indicates whether the same information was available either on the website or, if absent online, was communicated during at least 1 call. Thus, combined values reflect what a client would encounter after reviewing the website and contacting the center if needed.

### Website review and calls

On 6/9/25, the senior investigators (KYX, DRA) independently extracted data from each center's website using a codebook-based tool covering how services were described (eg, indications, contraindications). From 7/1/25-9/3/25, 3 trained research assistants (SS, RL, LG), blinded to website review findings to keep call assessments independent, made scripted inquiries portraying adult clients with major depressive disorder on escitalopram 10 mg, a standard adult dose selected to represent a routine clinical scenario centers may encounter. Callers additionally disclosed either a history of alcohol use disorder (Script #1) or a family history of psychosis (Script #2), assigned in randomized order. In Script #2, the caller described a paternal grandmother with severe depression who had once reported hearing voices in a religious context but had never been evaluated by a psychiatrist. The 2 scenarios probed distinct ends of the psilocybin-relevant clinical spectrum: alcohol use disorder is a condition for which psilocybin has emerging therapeutic evidence,^25^ while psychosis is a commonly cited concern in psychedelic safety discussions.^6,26,27^ Each center was contacted in 2 waves by separate research assistants using distinct caller names and phone numbers, with up to 3 attempts per wave. Callers documented staff communication in field notes covering safety, eligibility, and cost (eg, callers asked whether they would need to stop escitalopram before receiving psilocybin). Scripts, caller training, fidelity, and detection-mitigation procedures appear in eMethods 1 and 2.

### Debriefing (member checking)

After preliminary analysis, all 22 centers in the analytic sample received an email describing the mystery shopper study and inviting them to participate in a separately consented follow-up interview lasting ∼45 minutes (4/2026; eMethods 1). During the interview, participants viewed aggregate results and preliminary themes by screen share; center-specific responses were not disclosed. Participants, including facilitators and service center administrators, confirmed, challenged, and contextualized themes from the mystery shopper phase.^22^ Five centers participated, all of which had been reached by phone. Feedback was not treated as superseding observed data.

### Analytic approach

Quantitative findings were analyzed using SAS 9.4 with the service center as the primary unit of analysis; we also summarized responses across all completed calls at the call level.

For general safety and program requirements, such as the age requirement, collection of psychiatric history, and contraindications involving lithium or active suicidal ideation, we assessed whether a client would encounter the information anywhere along the usual pathway from website review to telephone contact. A center was counted as disclosing an item if it appeared online or was mentioned during at least 1 call.

For individualized assessments, such as whether psilocybin might help the caller's depression, whether escitalopram should be continued, or whether the caller could proceed with services, we assessed consistency across completed calls. A center was classified as “yes” or “no” only when both completed calls returned the same clear response. As described in the Codebook (eMethods 2), responses were classified as “equivocal” when staff neither clearly affirmed nor declined, but expressed substantive uncertainty or contingent reservations (eg, “that would depend”). At the center level, a center was classified as equivocal/uncertain if either call produced an equivocal response or if the 2 calls returned conflicting responses, treating within-center inconsistency as itself a finding, since a client contacting the center twice could encounter that same variability. Candidacy (“am I a good candidate?”) was assessed within each scenario rather than pooled across scripts, as the 2 calls posed clinically distinct questions.

Qualitative analysis proceeded in 2 stages. First, candidate themes emerged from caller field notes, supplemented by impressions (tone, hesitation, repeated patterns) shared during weekly team meetings. After data collection, the senior investigators independently re-reviewed all field-note transcripts using the codebook and constant comparative methods to identify emergent patterns.^28^ Discrepancies were resolved by consensus, and themes were refined until no new patterns emerged. Second, once these themes had been developed, debriefing interview notes were analyzed separately from caller field notes. Debriefing interviews served as member-checking, contextualizing themes from calls through staff perspectives on regulatory, economic, and operational dynamics not directly observable through the calls themselves, including how screening practices evolved over time and emerging themes such as how cannabis-based business expectations shaped the financial viability of service centers.

## Results

### Study sample and data collection overview

The OHA directory listed 26 licensed psilocybin service centers at website capture (June 2025); 4 had closed by the start of mystery shopper calls, leaving 22 centers in the analytic sample. Closure was verified by removal from the OHA licensee directory, a disconnected or nonworking telephone number, or a website that was no longer available. Reached and unreachable centers did not differ on observable website characteristics (geographic region, website presence, overall quality, pricing disclosure). As shown in Figure 1, callers reached 15/22 centers (68.2%), yielding 27 completed calls across 2 waves (wave 1: *n* = 14; wave 2: *n* = 13); the remaining 7 centers (31.8%) were unreachable (rang without answer, reached voicemail without a return call, or connected to a nonworking or disconnected number) despite up to 3 attempts per wave. Of the 27 calls, 15 used Script 1 (alcohol use disorder) and 12 used Script 2 (family history of psychosis); 12 centers responded to both, enabling within-center comparison. Facilitators (rather than administrative staff) answered 25/27 calls (92.6%), and at most centers where staff identified themselves, the same individual responded across waves (84%).

**Figure 1.**
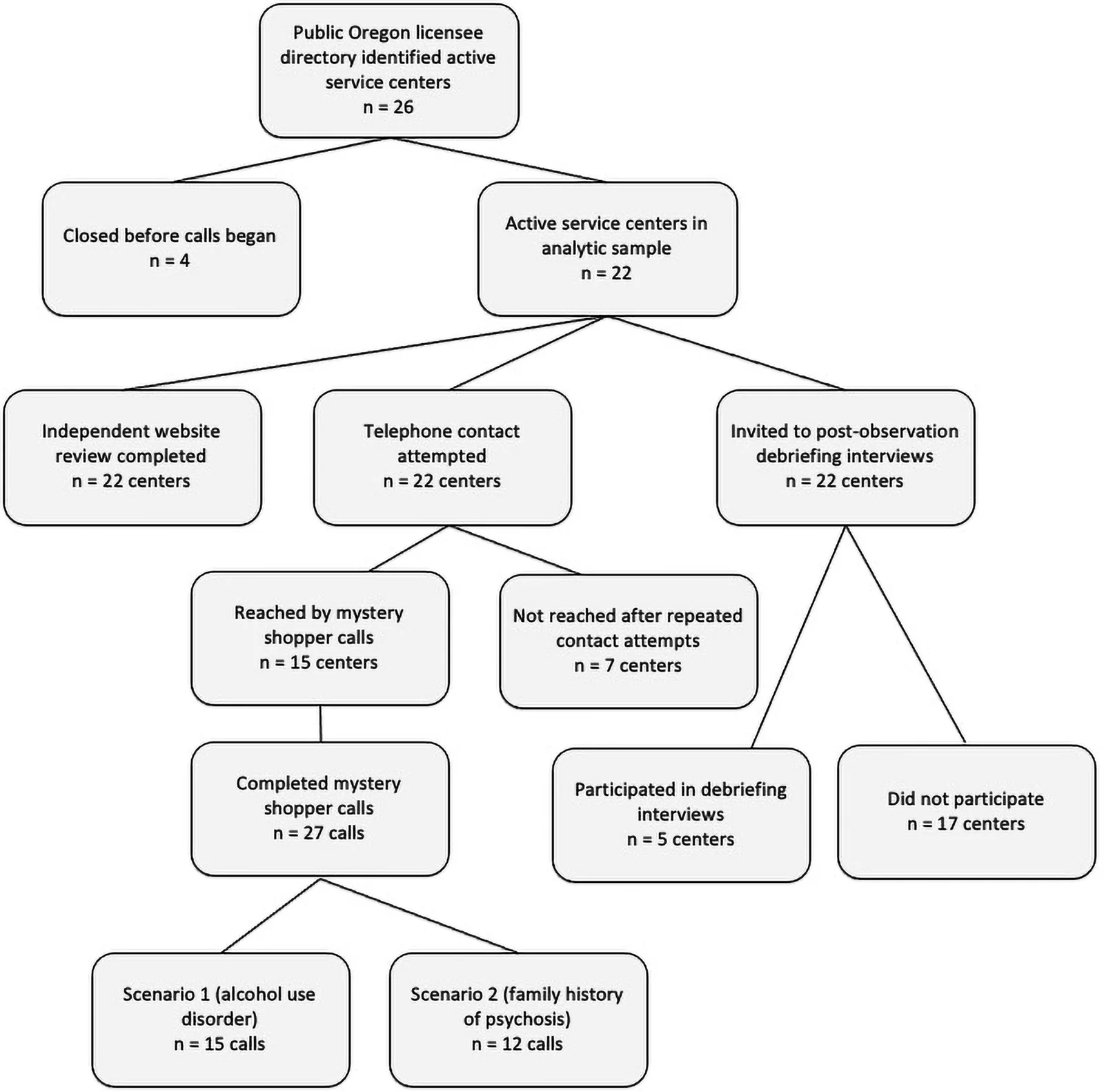
Summary of data collection for mystery shopper audit of Oregon psilocybin service centers.

Results are reported as proportions and summary statistics with the number of reached service centers (*n* = 15) as the unit of analysis. Comparisons across the 3 research assistants and the 2 scripted scenarios revealed no differences in safety, eligibility, or cost-related responses (eTable 1). Website and call results are presented together below across 3 domains (safety, eligibility, and cost and access).

### Safety

Communication of OHA safety requirements was more explicit during calls than on websites. When information obtained on websites was followed by a detailed inquiry on calls (reflecting a typical client pathway of reviewing the website and then contacting the center), information gaps observed online were substantially reduced. For example, 2 mental health-related contraindications were addressed more consistently during calls than on websites (Table 1). Specifically, among reached centers, lithium was identified as a contraindication on websites alone at 7 of 15 centers (46.7%) and during at least 1 call at 11 of 15 centers (73.3%); when both channels were considered together, 14 of 15 centers (93.3%) disclosed this contraindication. Active suicidal ideation was addressed by staff during at least 1 call at 13 of 15 centers (86.7%), and was addressed at all 15 reached centers (100%) either online or during a call, compared with 9 of 15 (60.0%) on websites alone.

On websites, the most frequently cited indications were depression (81.8%), post-traumatic stress disorder/trauma (77.3%), and anxiety (77.3%), followed by substance use disorders (68.2%). When callers asked about SSRI management, no center recommended discontinuation. At 8/15 centers (53.3%), both calls recommended continuing the current SSRI. At the remaining 7/15 (46.7%), responses were less clear: some centers declined to advise given concerns about SSRI–psilocybin interactions, while at others, responses conflicted across calls (1 recommending continuation, the other declining to advise). During these initial screening calls, staff who declined to advise on escitalopram generally deferred to the caller's existing prescriber. Because the calls did not proceed to formal preparation or evaluation, we could not determine whether centers would later involve a physician. When callers asked whether psilocybin could help with depression, 12/15 centers (80.0%) responded affirmatively across all calls; 3 centers (20.0%) returned equivocal responses (eg, “can’t give you…guarantees”). No center dismissed psilocybin as ineffective, nor did they offer immediate dosing without further screening.

No center permitted unsupervised or take-home dosing. Age requirements (21+) were posted on websites at 12 of 15 reached centers (80.0%) and confirmed during at least 1 call at 7 of 15 (46.7%); when both channels were considered together, 14 of 15 centers (93.3%) communicated the age requirement.

### Eligibility

Across both scenarios, centers generally described psilocybin eligibility as possible but contingent on further evaluation (eTable 2). Among the 15 centers reached on Script 1, 12 (80.0%) indicated that psilocybin could be appropriate for individuals with stable, sustained sobriety, with the remainder offering more hesitant responses; staff raised active drinking as disqualifying only after callers disclosed a history of alcohol use disorder, but no center described it as an absolute disqualification. Among the 12 centers reached on Script 2, 7 (58.3%) said the caller could potentially proceed if the family history of psychosis was clarified during intake. The remaining 5 (41.7%) identified the family history as a concern but would not say by phone whether the caller could proceed. Centers that discussed the family history disclosure also stated, without prompting, that a personal diagnosis of psychosis would be disqualifying under Oregon rules.

When callers asked whether they were potential candidates, responses were similarly consistent across scripts. At 9 of 15 centers on Script 1 (60.0%) and 8 of 12 centers on Script 2 (66.7%), staff indicated the caller could likely proceed pending further screening and intake evaluation (eg, “we’d go through it more carefully at intake”). The remaining centers were more hesitant, expressing reservations about whether the caller's history was appropriate for their setting without explicitly declining (eg, “not sure we’d be the right fit”). No center told the caller they were ineligible during the phone call.

### Cost and access

The median estimated total cost for a psilocybin session (preparation, dosing, integration) was $1900 (range, $850–$3000). The median time to first successful contact was 2.0 days (range, 1-8.5), and the median time to a scheduled intake appointment was 9.25 days (range, 5-30). No center accepted insurance, all reached centers offered some form of discount or sliding scale, and 4/15 (26.7%) accepted Health Savings Account payments. All reached centers confirmed that out-of-state clients could access services, with several noting anecdotally that out-of-state demand at times exceeded local demand.

### Themes from call field notes and debriefing interviews

Analysis of mystery shopper field notes and debriefing interviews identified 5 themes that contextualize the findings, shown in Table 2.

**Table 2.** Qualitative themes from debriefing interviews with Oregon psilocybin service center staff.

Theme	Representative observation or quotation
**Theme 1. Safety evaluations were treated as non-negotiable, individualized, and shaped by accumulated experience**	*"If you would have done this [study] in the first 6 months of this being open, the data would not be as nice. There is so much that I learned over the first 50 people that I sat with."*
	*"We always try to mitigate people's expectations, and I think that's part of what a facilitator should be doing."*
	*"There's a decent percent of people in this that grew up in the war on drugs era… and they won’t do anything illegal. They come here because it's a legal way to do it."*
	*"We take people who are on the straight and narrow…maybe 40% to 50% have never touched psychedelics before, and they want to do it in a safe and responsible way."*
	*“Our service center goes above and beyond the bar the OHA has set for us, and many other centers do as well. When we first saw the OHA guidelines, we thought “this is really restrictive, but in other ways it could be better.””*
**Theme 2. Unsupervised use was uniformly prohibited**	*"We encounter it quite a bit, people reaching out and asking to purchase mushrooms for personal use or to self-treat. It's always a no. We don’t want to jeopardize our license, and it's not within our ethics of client safety."*
	*"It's Oregon. If someone really wanted mushrooms, are they really going to come and pay me $100, or are they going to buy it for $30 underground?"*
	*“At our center, there is a safe room where product is stored that has a metal-safe door with complete inventory. Anytime product is consumed or wasted out, it must be reported on the OHA website along with amount of payment and date…”*
**Theme 3. Defining “therapy” within a wellness-based model remained unresolved**	*"I think the state folks are well-meaning and pioneers. It all has to evolve. They held things super strict and said we couldn’t say therapy [in the beginning]."*
	*"We always let clients interested in seeking services know that it is not a silver bullet, and it is not going to cure everything. And, it is a tool in a toolkit for general wellness and health."*
**Theme 4. Centers reported ongoing financial strain**	*"I’m never getting my investment back, it's gone. Now I just want to help as many people as possible. If I can continue doing this until I’m 90, that will be a life well lived."*
	*"And are we making that? Not really. I don’t know many service centers that are really ‘crushing it.'"*
	*"For facilitators, it costs 1k a year to maintain license. For service centers, it costs 10k a year. There is nothing between that."*
	*“Getting licensed as a facilitator is the most expensive application process I’ve ever witnessed… They are talking about doubling fees come June 2026, and there is no way we would be able to stay open.”*
	*“The survival rate is quite low—we don’t want this to become a failed system. If we fail our community in this because of finances, it will be devastating. These services have really helped people, and because of finances and accessibility, we can’t extend that net out further.”*
**Theme 5. Viability was constrained by market forces and misalignment with cannabis-derived business models**	*"We are currently going through a leveling process where a lot of early adopters and investors [from the cannabis industry] came in and thought they could use that same model with psilocybin, but since it's not a dispensary model, there is no medical use or recreational licensing, so there is no purpose to use cannabis as a model for that."*
	*"There were a lot more people [from the cannabis industry] who were here when the service centers first launched, and the business is a lot slower now. The only people that are left are the people that just want to see people get better."*
	*"Our service center cannot be based within 1000 feet from a school. It's hard to find somewhere that's drivable that can support a service center."*

#### Safety evaluations were non-negotiable and shaped by individualized assessment and experience

On mystery shopper calls, safety screening was consistently described as a non-negotiable step, reflected in deferral of medication decisions to prescribing clinicians and insistence on further evaluation before any dosing.

During debriefing, service center staff added that the cautious screening practices observed during the audit had developed through experience during the program's early years. Several suggested that an audit conducted closer to program launch might have found less consistent screening. As 1 facilitator remarked, “If you would have done this [study] in the first 6 months of this being open, the data would not be as nice. There is so much that I learned over the first 50 people that I sat with.” Staff also described their clientele as including substantial numbers of risk-averse clients: “There's a decent percent of people in this that grew up in the war on drugs era…They come here because it's a legal way to do it.” Across interviews, caution was described as central to what regulated services offer, with careful screening positioned as part of what distinguishes licensed centers from the underground market.

#### Unsupervised use was uniformly prohibited within a tightly regulated model

On mystery shopper calls, staff uniformly reinforced OHA prohibitions on take-home dosing, framing services as “serious” work rather than recreation. Several staff indicated they would report knowledge of unsupervised use outside licensed centers, but did not identify the person or agency they would contact. Debriefing interviews noted that requests for take-home psilocybin were common. One staff member framed this in the context of Oregon's broader market: “It's Oregon. If someone really wanted mushrooms, are they really going to come and pay me $100, or are they going to buy it for $30 underground?”

#### The status of “therapy” is unresolved at both the regulatory and practice levels

Whether psilocybin services constitute “therapy” is contested in Oregon's regulations and in national discussions of psychedelic care. On mystery shopper calls, this ambiguity surfaced: while many described their work as “non-directive facilitation,” terminology used in OHA regulations (OAR 333-333-5200), staff nevertheless described the boundary between facilitation and therapy as a “gray area,” particularly when discussing preparation, relationship-building, and integration sessions that resembled therapeutic processes. Debriefing interviews suggested that restrictions on clinical language have eased since the program's launch. As 1 participant noted, “I think the state folks are well-meaning and pioneers. It all has to evolve. They [OHA] held things super strict and said we couldn’t say therapy [in the beginning].” Staff also emphasized restraint in framing psilocybin's benefits, for example describing it as “not a silver bullet” and “a tool in a toolkit for general wellness and health.”

#### Centers reported ongoing economic strain, reflecting underlying financial fragility in the service model

On mystery shopper calls, staff described pricing in terms of facilitator labor, physical space, the cost of psilocybin, and training and licensing requirements.^29^ Many emphasized a willingness to adjust prices to meet clients' financial circumstances, encouraging callers to discuss costs directly with facilitators and referencing “social equity” programs (eg, sliding scale fees, scholarships). Debriefing interviews illustrated concerns about the financial viability of the current model. One staff member stated, “I’m never getting my investment back, it's gone. Now I just want to help as many people as possible.” Another offered a sobering assessment of the regulatory cost structure: “For facilitators, it costs 1k a year to maintain license. For service centers, it costs 10k a year. There is nothing between that.”

#### Viability was constrained by market forces and misalignment with cannabis-derived business models

Participants described early investors entering the market with expectations shaped by Oregon's higher-volume cannabis industry, despite a psilocybin model built around individualized screening and lengthy supervised sessions, limiting throughput and constraining revenue. As 1 interviewee put it, “There were a lot more people [from the cannabis industry] who were here when the service centers first launched, and the business is a lot slower now. The only people that are left are the people that just want to see people get better.” Geographic restrictions compounded these challenges. As 1 participant explained, “Our service center cannot be based within 1000 feet from a school. It′s hard to find somewhere that′s drivable that can support a service center.”

## Discussion

### Summary

Oregon's regulated psilocybin model appears more clinically cautious than critics of wellness-based access have feared, yet challenges with licensing costs and market forces make it expensive and difficult to scale. In this mystery shopper study, which traced the path a prospective client would follow from reviewing websites to contacting centers (Figure 2), staff engaged with psychiatric comorbidity in clinically nuanced ways not formally required by the regulatory framework. For example, staff worked through clinically ambiguous scenarios (family history of psychosis, current antidepressant use, alcohol use disorder) through follow-up questions, deferral of medication decisions to prescribing clinicians, and references to further evaluation before participation. Safety information sparse or absent on websites was often addressed during calls. Given concerns about unsafe self-directed use,^5,30,31^ every request to purchase psilocybin for unsupervised use was declined.

**Figure 2.**
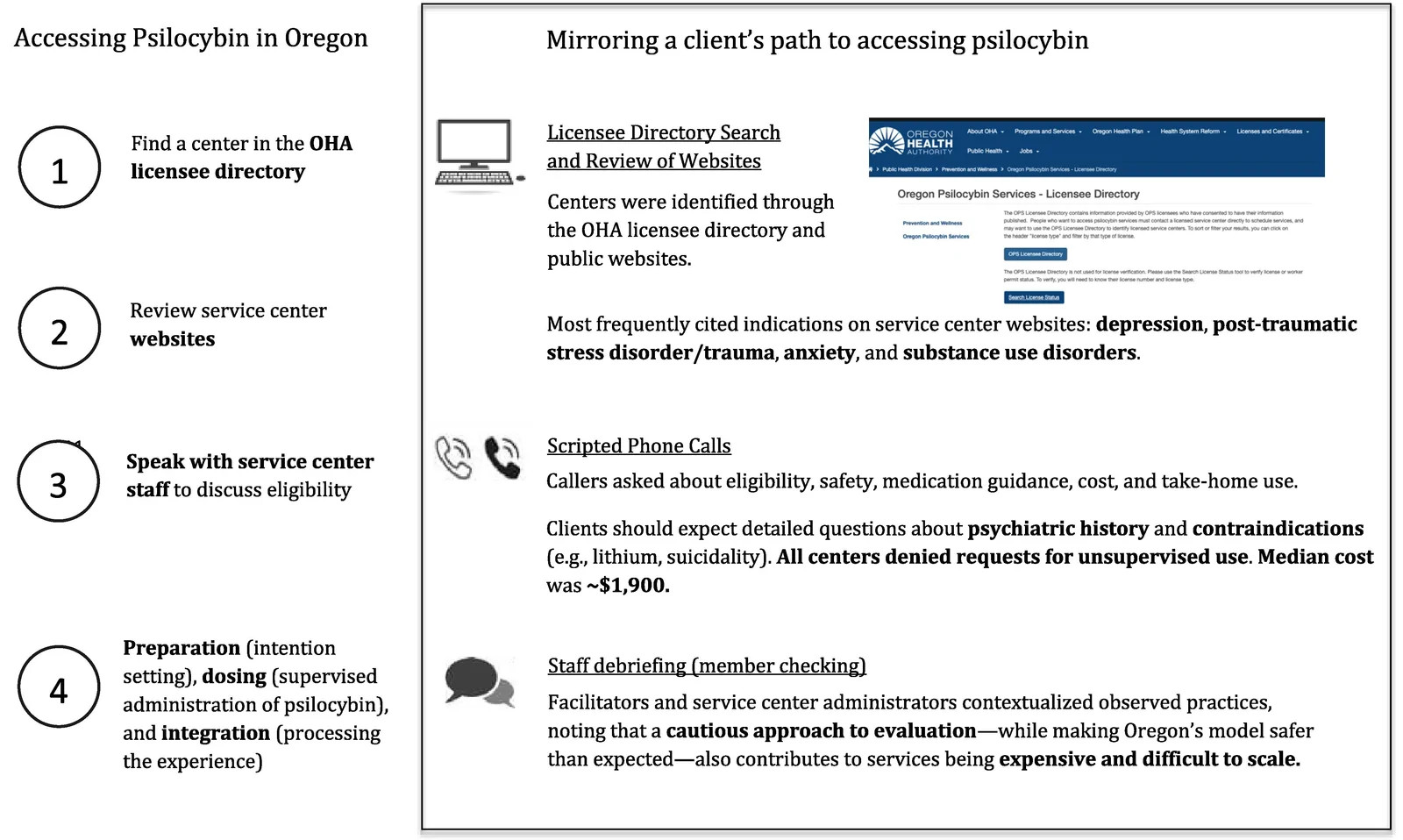
What prospective clients encounter when seeking psilocybin services in Oregon.

This caution extended to how centers described their services. Oregon's psilocybin regulations require clients be informed services “are not a medical or clinical treatment,”^32^ thus prohibiting facilitators from “diagnosing and treating physical or mental health conditions” and barring claims of “curative or therapeutic effects."^33^ Whether this holds in practice is less clear: recent scholarship has questioned whether the psychological support provided before, during, and after psilocybin administration can be meaningfully separated from psychotherapy.^31,34,35^ How this overlap should be understood is not settled, especially since preparation, dosing, and integration can occur outside clinical care, including in ceremonial contexts. Staff described this distinction as a “gray area,” often characterizing their work as “non-directive facilitation,” the term used in OHA regulations (OAR 333-333-5200), while also describing aspects of preparation and integration that overlapped with psychotherapy.

### Financial strain, Cannabis, and implications for policy

Despite reassuring safety findings, our data convey a system under financial strain. Four of the 26 originally licensed centers had closed by the start of data collection, consistent with reports of financial difficulty facing service centers.^36^ Seven centers (nearly 1/3 of the sample) could not be reached by phone after 3 contact attempts per wave; participants' accounts of slimly staffed operations competing with lengthy dosing sessions may also explain some of this. Because Oregon classifies psilocybin services as nonmedical, clients pay a median estimated $1900 out of pocket, and sliding-scale fees or scholarships may not eliminate the substantial upfront expense. Publicly available OHA administrative data^37^ show a substantial proportion of service denials categorized as “client ineligible” or “inconsistent with business model,” narrowing an already constrained revenue base.^38^ Among centers reached, intake appointments were often available within days, a pattern that may reflect provider capacity in excess of clients' ability to pay out of pocket.

These financial constraints may reflect a mismatch between expectations carried over from Oregon's cannabis market and the structure of supervised psilocybin services. Oregon's system aims to be budget-neutral to OHA, and as such, the program relies on costly license fees for centers and facilitators. Because clients in this model typically undergo only a small number of supervised sessions, centers cannot rely on the repeat-purchase volume that supports cannabis dispensaries, a constraint cannabis industry investors may have underestimated, alongside costly licensing fees and the labor-intensive nature of facilitation.^29^ As 1 staff member put it, early adopters from the cannabis industry “thought they could use that same model with psilocybin, but since it′s not a dispensary model, there is no medical use or recreational licensing.”

Prior commentary has warned that state-regulated psilocybin could follow the path of cannabis toward higher-volume retail sales.^4,12^ Our data do not suggest that shift was underway during the study period: participants described cannabis-linked investors leaving the market, while remaining centers continued to operate within Oregon's supervised-service model, though we cannot determine whether this reflects the regulatory structure or the limited profitability of the market.

Despite these divergent business models, Oregon's cannabis and psilocybin programs may nonetheless be converging under a single regulator. In summer 2026, OHA announced that the psilocybin services program will merge with the state's medical cannabis program (tentatively planned for September 2026), alongside a proposal to double facilitator and service center licensing fees.^39^ One interviewee had already flagged the latter: “Getting licensed as a facilitator is the most expensive application process I’ve ever witnessed… They are talking about doubling fees come June 2026, and there is no way we would be able to stay open.”

Taken as a whole, these economic constraints have implications for who is accessing psilocybin services. Although Oregon's program was designed with statewide access in mind, several centers described serving large numbers of clients from outside the state and internationally, suggesting that the client base may extend beyond the population OHA's mission was designed to reach.^40,41^ This was consistent with statewide OHA data from 2025, in which 32.6% of clients reported living outside Oregon, including 29.5% elsewhere in the United States and 3% outside the country.^37,42^ This pattern may reflect a preference among some individuals for regulated environments over informal settings,^43^ but it also suggests that the current model favors those with the resources to travel and pay out of pocket.^44,45^ Existing data show that the client base skews toward higher-income individuals relative to the general Oregon population,^37^ a pattern consistent with a median ∼$1900 price point.

### Limitations and strengths

Limitations are noted. First, these findings are preliminary and descriptive, reflecting 22 centers in 1 state in a rapidly evolving policy environment. Second, the small number of centers and repeated-contact design raised the possibility of recognition, though we mitigated this by switching callers and phone numbers between waves, and no indication of suspected repeat contact emerged during post-call debriefings. Third, centers unreachable by phone or closed during data collection are not represented in the call data. Fourth, our findings reflect first-contact communication and screening rather than the quality of subsequent intake, preparation, dosing (eg, decisions about micro vs macrodose), or integration sessions, including whether these were delivered remotely or in person. The calls did not capture detailed screening that centers conduct during intake or preparation, so initial responses by phone may not reflect the thoroughness of subsequent evaluation.

Fifth, our scripts are limited to selected clinical scenarios: depression, alcohol use disorder (which has the strongest evidence of efficacy among psychedelic-assisted treatments for substance use disorders^25^), and a possible family history of psychosis. Centers may screen, counsel, and price differently for other presenting conditions, and these findings are therefore an initial assessment rather than a comprehensive account of practices across indications. Finally, the OHA licensee directory lists only consenting licensees; while cross-checks with stakeholders familiar with Oregon's psilocybin service landscape did not identify additional active centers, the possibility that some were not listed cannot be excluded.

This study has notable strengths. It provides the first independent assessment of how the US's first regulated psilocybin service system functions in practice. The convergent design (integrating website review, calls, and debriefing interviews) captures the communication environment prospective clients encounter and triangulates observed practices with staff perspectives.

## Conclusions

Oregon's early experience with state-regulated psilocybin services suggests that a supervised, nonmedical model can support cautious screening of clients with psychiatric comorbidity. Our data show that clients interested in psilocybin can begin by finding service centers through the OHA directory, and that contacting centers directly typically yields answers to safety-related questions left unclear on websites (Figure 2). Clients should anticipate screening and questions about medications and contraindications rather than immediate access or unsupervised use, along with substantial out-of-pocket costs that are bound to increase if clients need repeat dosing. As other states consider similar models, our data suggest that Oregon's centers have struggled financially despite reassuring safety findings. Given that the program aims to be budget-neutral to OHA, with licensing costs falling on centers and facilitators and reflected in the prices clients pay, states designing such programs may need to consider how funding structures shape costs to clients, independent of the delivery model itself.

## Supplementary Material

qxag231_Supplementary_Data
