# Supplementary material for: “Am I a candidate for magic mushrooms?” A mystery shopper study of Oregon psilocybin service centers
Source: Health Aff Sch. 2026 Aug 27;4(10):qxag231. doi: 10.1093/haschl/qxag231 (PMC13659005; doi:10.1093/haschl/qxag231)
Supplement: Supplementary file 1 [file qxag231_supplementary_data.zip › 08.15.2026 supplement.docx]

### eMethods 1

IRB Approval

The Washington University Institutional Review Board approved the study, including a waiver of prospective informed consent for the covert observation phase (website review and mystery shopper telephone calls). To minimize burden on participating centers, callers limited each contact to a brief inquiry, did not request or schedule intake appointments, did not engage staff in extended conversation beyond what was needed to capture the prespecified communication domains, and made no more than three contact attempts per center per wave.

### Mystery Shopper Call Scripts

Both scripts portrayed the same baseline character: an adult prospective client with major depressive disorder currently taking escitalopram 10 mg, considering psilocybin services for depression. The two scripts differed only in one disclosure made midway through the call. In Script 1, the caller disclosed a history of alcohol use disorder with sustained sobriety, a condition for which psilocybin has emerging therapeutic evidence and a plausible reason a real client might seek services. In Script 2, the caller disclosed a family history of psychosis in a second-degree relative, specifically a paternal grandmother who had severe depression and had once reported hearing voices in a religious context but had never been evaluated by a psychiatrist. This history was intentionally uncertain. A personal history of active psychosis would require exclusion under Oregon rules and would leave little opportunity to observe how centers responded to an ambiguous safety signal, whereas an unverified family history falls within a recognized contraindication category in psychedelic screening guidelines while remaining clinically nuanced. Scripts were piloted with stakeholders familiar with Oregon’s psilocybin services to ensure realistic clinical framing and adequate specification of branching responses to anticipated staff questions.

Each script followed a common structure. Calls opened with a request to speak with a facilitator about psilocybin services. The caller then made a baseline disclosure of depression and current SSRI use, followed by the risk-relevant disclosure: alcohol use disorder history in Script 1 or family history of psychosis in Script 2. The caller then moved through standardized probes covering eligibility, safety considerations, candidacy for services, cost, and appointment availability. If staff asked clarifying questions about psychiatric history, medication, or motivation for seeking services, callers responded using pre-specified branching responses. Calls closed with the caller thanking staff and indicating they would follow up; no appointment was booked. Script order was randomized across waves so that each center received Script 1 in one wave and Script 2 in the other.

Codebook

The research team included investigators familiar with Oregon's psilocybin regulatory environment through prior engagement with OHA-licensed facilitators and service centers. None were employed by such centers, resided in Oregon or Washington, or had patients treated through these programs. This composition provided sufficient contextual familiarity to design a credible scripted protocol while maintaining geographic and professional independence consistent with audit study methodology.

We developed an a priori codebook before data collection. The codebook drew on Oregon Administrative Rules governing psilocybin services, which specify that psilocybin services do not constitute medical or clinical treatment and restrict unsupported therapeutic claims; publicly available OHA materials; and prior commentary identifying areas of potential consumer confusion.

Approximately 60 variables were defined across two data streams and three audit domains, with explicit operational criteria for every variable. Website variables (suffixed _web in the analytic dataset) covered safety and medical screening (including requirement for clients to provide psychiatric history; mentions of lithium and suicidal ideation being contraindications) and content on what psilocybin was being marketed for. Call variables (suffixed _ss in the analytic dataset) covered safety (e.g., alcohol-related and depression-related safety), eligibility (e.g., framing of whether clients were a candidate for psilocybin), and cost and access (e.g., total session cost, days to appointment).Website variables were coded as binary (yes/no) reflecting whether the relevant content was present online. Call variables for clinical-judgment and screening responses were coded using a three-level scheme (e.g., yes; equivocal/uncertain; no in response to “Am I a candidate?”) to distinguish clear endorsements from ambiguous staff responses. Continuous variables (e.g., total session cost) were recorded numerically. The codebook was refined iteratively as new response patterns emerged. The study team met regularly to assess whether definitions adequately captured observed responses, and iteration continued until coding categories were sufficient to represent observed variation. Once finalized, all prior records were re-reviewed for consistency.

Mystery Shopper Caller Training

Three research assistants conducted all mystery shopper calls using the application Burner® as trained members of the study team. None resided in Oregon or had personal experience as clients of psilocybin services in Oregon. Callers were assigned so that no center received calls from the same caller in both waves.

Before any live call, each caller completed a structured training sequence. Callers reviewed the codebook, both scripts, and branching response rules until familiar with the material. Each caller then completed five mock calls with a member of the study team playing the role of service-center staff. Mock calls included scenarios in which the staff member responded routinely, deviated from expected patterns, expressed suspicion about the caller's authenticity, or asked unscripted clinical questions. Callers were certified for live calls after completing at least two supervised mock calls reviewed by the principal investigators for script fidelity, pacing, and handling of unexpected responses.

Quality control during data collection consisted of three elements. After each call, the caller participated in a structured verbal debrief with a study investigator covering script delivery, any deviations, staff responses to unexpected questions, and any indications of caller detection. A second investigator independently reviewed field notes for completeness while details remained recent. The team met periodically throughout data collection to review patterns across calls and identify any drift in caller approach over time.

Because the small universe of licensed centers made detection a meaningful threat, we adopted several measures to reduce the probability that staff would recognize repeat contacts. A different research assistant conducted each wave of calls to a given center. Callers used different first names and different caller-ID phone numbers across waves. Calls within a wave were spaced across the data collection window rather than clustered in a short interval. Callers were instructed not to reference any prior contact or use language that might appear formulaic or rehearsed. Text messages were used only to schedule a live phone conversation; all study data were captured during the call itself.

Analytic Approach

*Unit of analysis.* The service center was the primary unit of analysis. We summarized findings describing what each service center, as an organization, communicated across calls and websites. The denominator for center-level analyses was the 15 reached centers, except for items specific to Script 2 (family-history-of-psychosis safety framing and Script 2 candidacy), where the denominator was the 12 centers reached on Script 2.

*Website-or-call safety disclosure.* For selected safety variables captured both online and during calls (psychiatric history required at intake, age requirement/verification, lithium contraindication, and active suicidal ideation), we constructed indicators reflecting whether a prospective client would encounter the information at any point in a typical access pathway. We treated website content as the initial exposure; if information was not present online, we then assessed whether it was communicated during at least one completed call. Centers were classified as affirmative if the information was available through either channel. These indicators were calculated among reached centers (n = 15) to allow direct comparison between website-only and combined website-plus-call disclosure.

*Clinical judgment variables.* For variables capturing a detailed clinical or evaluative recommendation (e.g., “will psilocybin help?”, SSRI continuation guidance, candidacy), we required concordance across calls before classifying a center as “yes” or “no.” A center was classified as “yes” only if all completed calls returned a clear “yes,” and as “no” only if all calls returned a clear “no.” Centers were classified as equivocal/uncertain in two circumstances: (1) any single call returned an equivocal response, or (2) calls to the same center returned conflicting clear responses (e.g., one call recommending continuation of an SSRI and another recommending taper). We treated this within-center inconsistency as substantively meaningful, as a prospective client contacting the center on different days could encounter that same inconsistency directly.

*Categories of incompleteness.* We distinguished four reasons a value could be absent from the dataset, only one of which we treated as missing data. Content was coded as not present when it was structurally absent from a website or a call (e.g., a website that did not mention age requirements or a call in which staff did not raise lithium). These values were treated as substantive findings rather than missing data. Content was coded as not asked when a variable was script-dependent and not relevant to the call (e.g., alcohol-use-disorder safety framing in Script 2 calls; family-history-of-psychosis framing in Script 1 calls; pregnancy, which was assessed only on websites). Not-asked values were excluded from the relevant denominator. Centers that could not be contacted were coded as not reached, and their calls were not analyzed. Content was coded as not disclosed when a topic was raised but staff declined to provide specific information; these responses were treated as a distinct form of non-response.

### Debriefing Interviews

After preliminary analysis of the website and mystery shopper data, all 22 service centers in the analytic sample received a written notification disclosing the study, summarizing preliminary findings, and inviting participation in a 45-minute structured debriefing interview. Participants provided informed consent before each interview. Participation in the debriefing phase was voluntary and required separate informed consent.

Five centers participated. Interviews were conducted via video conference by two investigators (XX, XX) in April 2026, and followed a structured four-part protocol. Each interview opened with a brief orientation covering study rationale, design, and methods. Investigators then presented preliminary quantitative findings and candidate themes via screen share. For each theme, participants were asked how the finding resonated with their experience, what was missing from the characterization, and what additional context would aid interpretation. Each interview closed with an open-ended question about what participants believed policymakers and the public should understand about Oregon's psilocybin services that the audit alone could not capture. Both investigators took field notes during each interview; interviews were not audio- or video-recorded.

Within one week of each interview, participants received a written document containing all candidate quotations attributed to their center, displayed as they would appear if used in publication. Participants could approve quotations as written, request edits for accuracy or clarity, or decline to have any specific quotation used. Only quotations that received explicit written approval were incorporated into the final manuscript, and all are presented anonymously, with no identifying information about the speaker, the center, or its location.

**eMethods 2A.** Codebook

| **Service center identifiers** | **agency** – This is the abbreviated name of **the service center**. The variable consists of text characters.  **enrolid** – This is an ID pertaining to each separate data point (secret shopper call + online webpage) or online webpage by itself (for those service centers that did not get a successful call). This is a number ranging from 1 to 34.  **agency_id** – Each agency has a unique ID number ranging from 1 to 22. Note that the dataset is currently presented in “**long form.”** If you want there to be one row per service center, that is possible (in SAS: Proc sort by “agency” and “agency_id”, then apply a data step that limits one row per service center via “if first.agency_id”). |
| --- | --- |
| **Website-derived variables** | **therapy_web** – This tells us whether the service center framed their services as following a medical model. 1 = clear yes,  2 = equivocal/ambivalent/it’s complicated  3 = clear no  Example of a 1:  "Psilocybin has also been shown to be helpful in addressing…[specific mental health conditions]."  Example of a 2:  "Psilocybin enables healing…"  (with no mention of medical language like “treatment”, “therapy”)  Example of a 3:  "_ is not a therapy center…  **process_web** – Do they describe the process online (prep, administration, integration)?  1 = yes, 3 = no  **consult_web** – Does the service center clearly specify that there is a need for consultation/intake interview before beginning preparation?  1 = yes, 3 = no  **govcomply_web** – Does the service center illustrate compliance with the Oregon Health Authority?  Examples include: “Intake forms are required 24 hours before the first dosing session” “Fully licensed by the Oregon Health Authority”  1 = yes, 3 = no  **underage_web** - Does the website clearly indicate that psilocybin is only allowed for people aged ≥21 years?  This may involve warnings on the webpage, pop-up menus, or mandatory checkboxes before filling out intake forms.  1 = yes, 3 = no  **med_web** – Does the website indicate evidence of a collaborative approach with medically licensed clinicians?  (For example, physicians on staff, or assistance with medical referrals.)  1 = yes, 3 = no  **allergies_web** – Do intake forms ask about allergies? Or are allergies discussed in any section of the website?  1 = yes, 3 = no  **psychhx_web** – Is it explicit on the website that psychiatric history is required to receive psilocybin (OHA-mandated forms)?  1 = yes, 3 = no  **scz_risk_web** – The website mentions psychotic disorders as a risk factor or asks about SCZ explicitly in pre-intake forms.  1 = yes, 3 = no  **bpad_risk_web** – The website mentions bipolar spectrum disorders as a risk factor or asks about BPAD explicitly in pre-intake forms.  1 = yes, 3 = no  **sud_risk_web** – The website mentions substance use disorders as a risk factor or asks about SUD explicitly in pre-intake forms.  1 = yes, 3 = no  **cv_web** – The website mentions cardiovascular conditions (including hypertension) as a risk factor or asks about cardiovascular conditions explicitly in pre-intake forms.  1 = yes, 3 = no  **sz_web** – The website mentions seizure disorders as a risk factor or asks about seizure disorders explicitly in pre-intake forms.  1 = yes, 3 = no  **lithium_web** – The website mentions receipt of Lithium as a risk factor or asks about lithium explicitly in pre-intake forms.  1 = yes, 3 = no  **medrec_web** – The website discusses the need to review psych med history (e.g., "Med list required for pre-eval", "Asks about SSRI, SNRI, mood stabilizer, anxiolytic, etc. and whether patient is open to tapering off").  1 = yes, 3 = no  **pregnancy_web** – Website inquires about pregnancy or breastfeeding.  1 = yes, 3 = no  **currentsi_web** – Website suggests that current suicidal ideation is a disqualifier or of concern.  1 = yes, 3 = no  **txanx_web** – Website frames psilocybin as potentially helpful for anxiety.  1 = yes, 3 = no  **txmdd_web** – Website frames psilocybin as potentially helpful for depression.  1 = yes, 3 = no  **txptsd_web** – Website frames psilocybin as potentially helpful for PTSD or trauma.  1 = yes, 3 = no  **txocd_web** – Website frames psilocybin as potentially helpful for obsessive-compulsive disorder (OCD).  1 = yes, 3 = no  **txterminal_web** – Website frames psilocybin as potentially helpful for terminal illness or end-of-life distress.  1 = yes, 3 = no  **txsud_web** – Website frames psilocybin as potentially helpful for substance use or addiction.  1 = yes, 3 = no  **txeating_web** – Website frames psilocybin as potentially helpful for eating disorders or disordered eating.  1 = yes, 3 = no  **txgrief_web** – Website frames psilocybin as potentially helpful for grief and bereavement.  1 = yes, 3 = no  **neurodiversity_web** – Website frames psilocybin as potentially helpful for ADHD, autism spectrum disorder, or broader neurodivergence.  1 = yes, 3 = no  **txrelationships_web** – Website frames psilocybin as potentially helpful for relationship improvement (e.g., couples therapy, gender-based support groups).  1 = yes, 3 = no  **txspiritual_web** – Website frames psilocybin as potentially helpful for spiritual growth or self-actualization.  1 = yes, 3 = no  **txcreative_web** – Website frames psilocybin as potentially helpful for creativity.  1 = yes, 3 = no  **transportation_web** – Website explicitly mentions that patients cannot drive after dosing.  1 = yes, 3 = no  **prelimintake_web** – Website states that preliminary intake form must be completed before dosing prep.  1 = yes, 3 = no |
| **Secret shopper-derived variables** | **centerreached_ss** – This basically tells us whether the center was reached via secret shopper call or not.  1 = yes, 3 = no.  **data_ss** – Is this a secret shopper data point?  1 = yes, 3 = no (for the service centers that we could not reach, last 7 rows of the spreadsheet)  **samestaff_ss** – During the secret shopper call, was it the same staff member each time?  1 = yes, 3 = no, 999 = if only reached once (i.e., Gather)  **round_ss** – Which round of the secret shopper study does this data point pertain to?  1 = round 1, 2 = round 2, 999 = if datapoint is for a service center that was not reached for SS  **script_ss** – Which script number is this?  1 = SCRIPT 1: Is psilocybin safe with a history of alcohol use disorder?  2 = SCRIPT 2: Is psilocybin safe with a potential family history of schizophrenia?  999 = if datapoint is for a service center that was not reached for SS  **facilitator_ss -** To the best of your knowledge, did you reach the facilitator or owner? (as opposed to receptionist)  1= Facilitator or owner  3= Receptionist or other staff member  **investigator_ss** – Who was the investigator doing the call?  1 = RA 1  2 = RA 2  3 = RA 3j  999 = if datapoint is for a service center that was not reached for SS  **attempts_ss** – Number of times attempted before reaching the service center.  1 = once  2 = twice  3 = 3 or more times  999 = if datapoint is for a service center that was not reached for SS  **mode_ss** – How did you reach the service center? Some may be reached via just phone calls; others may require texting.  1 = phone  2 = text  3 = both  999 = if datapoint is for a service center that was not reached for SS  **attemptdays_ss** – Numeric variable for number of days it took to reach a staff member  999 = if datapoint is for a service center that was not reached for SS  **alcsafe_ss** – Is psilocybin safe with a history of alcohol use disorder?  1 = yes  Example: “There's evidence now that psilocybin helps people with alcohol use disorder….”  2 = equivocal/ambivalent/it’s complicated  Examples: “No research to suggest one way or the other.”  3 = no  999 = if data point is for a service center that was not reached for SS  **sczsafe_ss** – Is psilocybin safe with a potential family history of schizophrenia?  1 = yes  Example: “Doesn't worry me too much.”  2 = equivocal/ambivalent/it’s complicated  Examples: “I cannot comment on that.”  3 = no  999 = if datapoint is for a service center that was not reached for SS  **mdd_ss** – Will psilocybin help with depression?  1 = yes  Examples: “That is what psilocybin is best for….”  2 = equivocal/ambivalent/it’s complicated  Examples: “I can't give you any legal guarantees.” “ not allowed to make claims.”  3 = no  999 = if datapoint is for a service center that was not reached for SS  **si_ss** – Is psilocybin safe for people who have struggled with suicidal thoughts in the past?  1 = yes  Examples: “as long as you're not actively suicidal now.”  2 = equivocal/ambivalent/it’s complicated  Examples: “depends…  3 = no  999 = if datapoint is for a service center that was not reached for SS  **currentsi_ss -** Did the service center staff member spontaneously bring up that active SI is a contraindication? The callers did NOT explicitly ask any staff member about policies surrounding active SI. However, while coding the field notes, we noticed that the majority of centers articulated, ***unprompted***, that active SI was a no-no, and so we added this item to the dataset. We did not sense any ambivalence when it came to this topic, and so we only have two options- 1 and 3.  1= yes  3 = no  **lithiumsi_ss -** similar to currentsi_ss, the callers did not explicitly ask about lithium being a contraindication. While coding the field notes, we saw that service centers were articulating, ***unprompted,*** that they would not take people who were prescribed lithium. We did not sense any ambivalence when it came to this topic, and so we only have two options- 1 and 3.  1= yes  3 = no  **ssri_ss** – Do you require or strongly recommend that I stop my medications (‘Lexapro 10’) to get psilocybin?  1 = yes (note: no service center recommended this unequivocally)  2 = equivocal/ambivalent/it’s complicated  Examples: “That would be a question between you and your healthcare provider…”  3 = no  Examples: “I suggest staying on it.”  999 = if datapoint is for a service center that was not reached for SS  **psychmd_ss** – Do you require or strongly recommend prior evaluation by a psychiatrist before starting psilocybin?  1 = yes  Examples: “It is definitely recommended.”  2 =equivocal/ambivalent/it’s complicated  Examples: “It would be good for them to know that their patients are using psychedelics, but …no, you don’t need to….”  3 = no  Examples: “We don’t require it.”  999 = if datapoint is for a service center that was not reached for SS  **medclear_ss** – Do you require or strongly recommend medical clearance with a clinician?  1 = yes (no center was forceful in recommending medical clearance or that you call your PCP…)  2 = equivocal/ambivalent/it’s complicated  Examples: “It would be in your best interest if you have something seriously going on that we should know about. But no, we don’t require clearance.”  3 = no  Examples: “But no, we don’t require clearance.”  999 = if datapoint is for a service center that was not reached for SS  **underage_ss** – Asked if underage  1 = yes, 3 = no  999 = if datapoint is for a service center that was not reached for SS  **apptdate_ss** – Number of days to make an initial intake appointment  999 = if datapoint is for a service center that was not reached for SS  **prepsetting_ss** – How are prep sessions done?  1 = virtual  2 = in person  3 = both  999 = if datapoint is for a service center that was not reached for SS  **oos_ss** – Are out-of-state clients allowed?  1 = yes  3 = no  999 = if datapoint is for a service center that was not reached for SS  **home_ss** – Can I bring psilocybin home?  1 = yes  3 = no  999 = if datapoint is for a service center that was not reached for SS  **candidate_ss** – Am I a potential candidate for psilocybin?  1 = likely  Examples: “No red flags have come up for me at all.”  2 = equivocal/ambivalent/it’s complicated  Examples: “I'm not gonna give you an opinion.”  3 = no  999 = if datapoint is for a service center that was not reached for SS  **insurance_ss** – Do you accept insurance?  1 = yes  3 = no  **cost_ss** – What’s the cost for everything (i.e., prep, meds)? (if provided range of costs, then we took the average)  999 = if datapoint is for a service center that was not reached for SS  **discount_ss** – Are discounts available? (could include sliding scale, scholarship, group discounts, HSA options, pay it forward/installments). Note that the question was originally phrased as ‘do you accept sliding scale?’ While coding field notes, we noticed that staff members discussed, unprompted, other forms of discounts beyond just sliding scale (i.e., “scholarship” applications; group sessions being cheaper; potential HSA payments). For this reason, we broadened the former “sliding scale” question to include other forms of discounts.   1 = yes  3 = no  999 = if datapoint is for a service center that was not reached for SS  **Group_ss -** is group session discounts available? We did not explicitly ask about discounts for people interested in being dosed in a group format. Because this kept coming up ***unprompted***, we created a separate question on group treatment discounts.  1=yes  3=no  **HSA_ss -** have some clients paid via HSA? Similar to above, we did not explicitly ask about clients using HSAs to pay for psilocybin. Because this kept coming up ***unprompted***, we created a separate question on HSAs.  1=yes  3=no  **therapy_ss**  1 = yes  “That is absolutely what we do.”  2 = equivocal/ambivalent/it’s complicated  “Yes and no…gray zone…”  3 = no  “ not really allowed to call it therapy.”  999 = if datapoint is for a service center that was not reached for SS |

**eMethods 2B**. Scripts.

Phone Script 1 – History of Alcohol Use Disorder

Hello- I found this number from the State of Oregon’s healing center directory. I had a few questions about your services and whether I would be a candidate for psilocybin treatment. Can I speak to someone who could answer them?

When is your next available appointment? [IF UNABLE TO GET AN EXACT DATE, ASK FOR AN ESTIMATE]

Is that appointment for intake/screening or preparation? [Type of appointment]

Can I do my prep and integration sessions virtually?

- If no appointments available: ‘Can you refer me somewhere else?’

I might be moving to Vancouver, Washington in a few months. Would I still be able to get treatment if I’m not an Oregon resident anymore?

Do we have the option of taking psilocybin to use at home?

Do you accept insurance?

- [IF YES], What types of insurance do you accept? How much is the copay? Is that for everything, like prep, meds, and integration? Or is there a separate charge for prep, meds, and integration?
- [IF NO], THEN ASK: “How much is the cost? Is that for everything, like prep, meds, and integration? Or is there a separate charge for prep, meds, and integration?”]

Do you offer a sliding fee scale?

Do you offer psychedelic-assisted therapy? I hear that term a lot but don’t know what it means.

I’ve been struggling with depression for some time and am doing a little better lately. I still have some bad days and am wondering if psilocybin might help.

Would I need to stop my medications (‘Lexapro 10’) to get psilocybin?

I’ve also been struggling w/ alcoholism for some time. This makes me a bit nervous about psilocybin. Is it still safe with this history?

- [IF THEY ASK FOR MORE DETAILS ON ALCOHOL HISTORY, SAY:“I am 6 months sober. I am actually having no cravings at all right now and want to keep the momentum going. I vaguely remember reading somewhere that psilocybin might help with sobriety” ]

Do you know if psilocybin is safe for people who have struggled with suicidal thoughts in the past?

I haven’t seen my psychiatrist in a while and probably should. Do you require prior evaluation by a psychiatrist before starting psilocybin? Also, do I need medical clearance?

- [IF THEY HAVEN’T ALREADY SAID IF YOU ARE OR ARE NOT A CANDIDATE FOR TREATMENT]: Do you think I am a good candidate for psilocybin treatment?
- [IF THEY SAY YOU ARE NOT A GOOD CANDIDATE AT ANY POINT, ASK: Is there a way for me to become a better candidate? If not, what other treatments or alternatives would you recommend for me?]

Wow this is a lot of information. I need to think this through a little more. Thank you for your time. [END PHONE CALL.]

Phone Script Version 2 – Family History of Schizophrenia

Hello- I found this number from the State of Oregon’s healing center directory. I had a few questions about your services and whether I would be a candidate for psilocybin treatment. Can I speak to someone who could answer them?

When is your next available appointment? [IF UNABLE TO GET AN EXACT DATE, ASK FOR ESTIMATE]

Is that appointment for intake/screening or preparation? [Type of appointment]

Can I do my prep and integration sessions virtually?

- If no appointments available: ‘Can you refer me somewhere else?’

I might be moving to St. Louis in a few months for a job. Would I be able to access treatment there?

Do we have the option of taking psilocybin to use at home?

Do you accept insurance?

- [IF YES], THEN ASK: What types of insurance do you accept? How much is the copay? Is that for everything, like prep, meds, and integration? Or is there a separate charge for prep, meds, and integration?
- [IF NO], THEN ASK: “How much is the cost? Is that for everything, like prep, meds, and integration? Or is there a separate charge for prep, meds, and integration?”]

Do you offer a sliding fee scale?

Do you offer psychedelic-assisted therapy? I hear that term a lot but don’t know what it means.

I’ve been struggling with depression for some time and am doing a little better lately. I still have some bad days and am wondering if psilocybin might help.

Would I need to stop my medications (‘Lexapro 10’) to get psilocybin?

Schizophrenia might run in my family. This makes me a bit nervous about psilocybin. Is it still safe with this history?

- [IF THEY ASK FOR MORE DETAILS ON SCHIZOPHRENIA HISTORY, SAY: “Well…My grandma on my dad’s side struggled with a lot of mental health issues. I actually am not sure if it was schizophrenia. I mean, she had really bad depression and at one point was hearing voices. But she was also a very spiritual person and often heard voices when talking with God. I actually don’t think she ever sought treatment from a psychiatrist. ]

Do you know if psilocybin is safe for people who have struggled with suicidal thoughts in the past?

I haven’t seen my psychiatrist in a while and probably should. Do you require prior evaluation by a psychiatrist before starting psilocybin? Also, do I need medical clearance?

- [IF THEY HAVEN’T ALREADY SAID IF YOU ARE OR ARE NOT A CANDIDATE FOR TREATMENT]: Do you think I am a good candidate for psilocybin treatment?
- [IF THEY SAY YOU ARE NOT A GOOD CANDIDATE AT ANY POINT, ASK: Is there a way for me to become a better candidate? If not, what other treatments or alternatives would you recommend for me?]

Wow this is a lot of information. I need to think this through a little more. Thank you for your time.

[END PHONE CALL.]

**eTable 1.** Comparison of mystery shopper findings across the three research assistants who conducted calls

| **Variable** | **Caller 1**  **(n=10)** | **Caller 2**  **(n=8)** | **Caller 3**  **(n=9)** | **p-value** |
| --- | --- | --- | --- | --- |
| Age verification asked |  |  |  | *0.70* |
| Yes | 4 (40.0%) | 2 (25.0%) | 3 (33.3%) |  |
| No | 5 (50.0%) | 6 (75.0%) | 6 (66.7%) |  |
| *Missing* | *1 (10.0%)* | *0 (0%)* | *0 (0%)* |  |
| Lithium flagged as contraindication |  |  |  | *0.79* |
| Yes | 6 (60.0%) | 4 (50.0%) | 4 (44.4%) |  |
| No | 4 (40.0%) | 4 (50.0%) | 5 (55.6%) |  |
| Active suicidal ideation flagged |  |  |  | *0.51* |
| Yes | 8 (80.0%) | 5 (62.5%) | 5 (55.6%) |  |
| No | 2 (20.0%) | 3 (37.5%) | 4 (44.4%) |  |
| SSRI management |  |  |  | *0.11* |
| Continue (no taper) | 8 (80.0%) | 3 (37.5%) | 7 (77.8%) |  |
| Equivocal / uncertain | 2 (20.0%) | 5 (62.5%) | 2 (22.2%) |  |
| Depression: will psilocybin help? |  |  |  | *0.42* |
| Yes | 8 (80.0%) | 8 (100%) | 7 (77.8%) |  |
| Equivocal / uncertain | 2 (20.0%) | 0 (0%) | 1 (11.1%) |  |
| *Missing* | *0 (0%)* | *0 (0%)* | *1 (11.1%)* |  |
| Am I a candidate? (combined across scripts) |  |  |  | *0.16* |
| Yes | 4 (40.0%) | 6 (75.0%) | 7 (77.8%) |  |
| Equivocal / uncertain | 6 (60.0%) | 2 (25.0%) | 2 (22.2%) |  |
| HSA accepted |  |  |  | *0.59* |
| Yes | 2 (20.0%) | 1 (12.5%) | 3 (33.3%) |  |
| No | 7 (70.0%) | 7 (87.5%) | 6 (66.7%) |  |
| *Missing* | *1 (10.0%)* | *0 (0%)* | *0 (0%)* |  |
| Insurance accepted (No) | 10 (100%) | 8 (100%) | 9 (100%) | *—* |
| Out-of-state clients accepted (Yes) | 10 (100%) | 8 (100%) | 9 (100%) | *—* |
| Estimated cost, USD | $1,950 (850–3,000) | $1,425 (850–3000) | $1,900 (850–3000) | *0.84* |
| Days to first contact | 2.0 (1–14) | 1.0 (1–10) | 2.0 (1–7) | *0.17* |
| Days to intake appointment | 7.0 (2–30) | 10.5 (7–30) | 7.0 (3–21) | *0.65* |

**eTable 2.** Comparison of mystery shopper findings across the two scripted scenarios (Script 1: alcohol use disorder; Script 2: family history of psychosis)

| **Variable** | **Script 1**  **AUD (n=15)** | **Script 2**  **FHx psychosis (n=12)** | **p-value** |
| --- | --- | --- | --- |
| Age verification asked |  |  | *0.68* |
| Yes | 6 (40.0%) | 3 (25.0%) |  |
| No | 9 (60.0%) | 8 (66.7%) |  |
| *Missing* | *0 (0%)* | *1 (8.3%)* |  |
| Lithium flagged as contraindication |  |  | *0.70* |
| Yes | 7 (46.7%) | 7 (58.3%) |  |
| No | 8 (53.3%) | 5 (41.7%) |  |
| Active suicidal ideation flagged |  |  | *1.00* |
| Yes | 10 (66.7%) | 8 (66.7%) |  |
| No | 5 (33.3%) | 4 (33.3%) |  |
| SSRI management |  |  | *0.45* |
| Continue (no taper) | 11 (73.3%) | 7 (58.3%) |  |
| Equivocal / uncertain | 4 (26.7%) | 5 (41.7%) |  |
| Depression: will psilocybin help? |  |  | *0.58* |
| Yes | 13 (86.7%) | 10 (83.3%) |  |
| Equivocal / uncertain | 1 (6.7%) | 2 (16.7%) |  |
| *Missing* | *1 (6.7%)* | *0 (0%)* |  |
| Am I a candidate? (combined) |  |  | *0.99* |
| Yes | 9 (60.0%) | 8 (66.7%) |  |
| Equivocal / uncertain | 6 (40.0%) | 4 (33.3%) |  |
| HSA accepted |  |  | *0.65* |
| Yes | 4 (26.7%) | 2 (16.7%) |  |
| No | 10 (66.7%) | 10 (83.3%) |  |
| *Missing* | *1 (6.7%)* | *0 (0%)* |  |
| Insurance accepted (No) | 15 (100%) | 12 (100%) | *—* |
| Out-of-state clients accepted (Yes) | 15 (100%) | 12 (100%) | *—* |
| **Continuous variables — median (range)** | | | |
| Estimated cost, USD | $1,900 (850–3000) | $1,700 (850–3,000) | *0.70* |
| Days to first contact | 2.0 (1–14) | 1.0 (1–10) | *0.10* |
| Days to intake appointment | 7.0 (7–30) | 7.0 (2–30) | *0.83* |
